# Vitamin C Levels in Different Organs of Bat Species from Different Food Groups

**DOI:** 10.3390/life12122121

**Published:** 2022-12-15

**Authors:** Diego Antonio Mena Canata, Mara Silveira Benfato, Francielly Dias Pereira, María João Ramos Pereira, Pabulo Henrique Rampelotto

**Affiliations:** 1 Biophysics Department, Universidade Federal do Rio Grande do Sul, Porto Alegre 91501-970, Brazil; 2 Graduate Program in Cellular and Molecular Biology, Universidade Federal do Rio Grande do Sul, Porto Alegre 91501-970, Brazil; 3 Graduate Program in Animal Biology, Universidade Federal do Rio Grande do Sul, Porto Alegre 91501-970, Brazil; 4 Graduate Program in Biological Sciences: Pharmacology and Therapeutics, Universidade Federal do Rio Grande do Sul, Porto Alegre 91501-970, Brazil

**Keywords:** ascorbic acid, bats, nectarivore, frugivore, insectivore, vampire bat

## Abstract

Unlike most animals, most bats cannot synthesize vitamin C endogenously. Consequently, this vitamin must be obtained from the diet. Among the bat species, there are several food groups, such as frugivorous, nectarivorous, insectivorous, and hematophagous. In this work, we measured and compared vitamin C levels in different organs of four species of bats, all collected in southern Brazil. When analyzing and comparing the levels of vitamin C in the four bat species, (regardless of the organ), no significant differences were observed. However, when analyzing and comparing the levels of vitamin C in the four organs (regardless of the species), significant differences were observed, with the highest concentrations in the heart, followed by the liver and brain, while the lowest concentration was measured in the kidneys. Additional differences in the levels of Vitamin C were only observed when each organ was analyzed according to the species/diet. These results indicate a high degree of metabolic homeostasis in bats despite the marked difference in the type of diet.

## 1. Introduction

L-ascorbic acid or vitamin C is an important nutrient necessary for a wide range of metabolic processes [1,2,3,4]. Although most organisms synthesize vitamin C, a limited number of mammalian species, primates of the suborder Haplorrhini (including humans and apes), and bats (most species) are deficient in their ability to synthesize this vitamin due to a lack of activity in the enzyme L-ascorbate gulonolactone oxidase (GULO), an enzyme that catalyzes the last step of biosynthesis. Consequently, vitamin C must be obtained from the diet, i.e., exogenously [5]. Among the bat species that do not synthesize vitamin C, there are several food groups, such as frugivorous, nectarivorous, insectivorous, and hematophagous.

*Glossophaga soricina* is a nectarivorous bat species distributed throughout South and Central America. It has a fast metabolism and can feed on the nectar of flowers and floral parts [6]. *Sturnira lilium* is a frugivorous bat species from South America (Brazil, Bolivia, Paraguay, Uruguay, and Argentina) that has a high preference for fruits from the Solanaceae family and a lower preference for fruits from the Moraceae, Piperaceae, and Bombanaceae families [7]. *Molossus molossus* is an insectivorous bat species distributed throughout South and Central America. This species can feed on a wide variety of insects but mainly prefers Coleoptera. The common vampire bat *Desmodus rotundus* is a small bat native to South and Central America and the only species that feeds on the blood of domestic cattle [8].

These different bat species with different food groups need to obtain vitamin C from their diet. While fruits and nectar have a high and medium content of vitamin C, respectively, and are both reasonably accessible to the frugivorous and nectarivorous bat species, what happens to the insectivorous and hematophagous bats with less access to this vitamin? In addition, how is vitamin C distributed among the key organs of the body? To answer these questions, the aim of this work was to measure and compare the levels of vitamin C in different organs of four bat species that cannot synthesize this vitamin. All samples were collected in southern Brazil, namely: *G. soricina* (nectarivorous), *S. lilium* (frugivorous), *M. molossus* (insectivorous), and *D. rotundus* (hematophagous).

## 2. Material and Methods

### 2.1. Animals and Samples Collection

Thirty-nine adult male bats were captured between the summer of 2018 and the winter of 2019 using dip nets, mist nets, or harp traps, depending on the type of shelter, in southern Brazil (Table S1). The bat species captured were *G. soricina* (*n* = 10), *S. lilium* (*n* = 10), *M. molossus* (*n* = 10) and *D. rotundus* (*n* = 9). Capture happened at the beginning of the night to ensure that all bats were fasted and so that food intake did not bias vitamin C levels. The animals were euthanized after capture by intraperitoneal injection with a combination of xylazine (10 mg/kg) and ketamine (60 mg/kg) to remove of all organs. Organs were frozen in liquid nitrogen immediately and stored at −80 °C for further analysis and testing.

### 2.2. Organ Processing

Brains, hearts, livers, and kidneys were manually macerated by Potter with a 30 mmol/L phosphate buffer, 120 mmol/L KCl, 0.201 mmol/L PMSF 150 µmol/L deferoxamine in pH 7.4 and centrifuged for 10 min, 14,000× *g*. The supernatant was aliquoted and frozen at −80 °C for later analyses and assays.

### 2.3. Vitamin C Assay

Vitamin C levels were measured by HPLC employing a reversed-phase column (SUPELCOSIL™ LC-18-DB HPLC column; 15 cm × 4.6 mm, 5 μm) using a mobile phase flow rate of 1 mL/min in 30 mmol/L monobasic potassium phosphate (pH 3.6) and methanol (9:1, *v*/*v*); samples were injected at a volume of 25 μL. The absorbance of the column effluent was monitored at 254 nm [9].

### 2.4. Statistical Analysis and Data Normalization

To test for significant differences among sample grouping, nonparametric permutation-based multivariate analysis of variance (PERMANOVA) with 999 permutations, followed by a Bonferroni-corrected PERMANOVA pairwise comparison, was performed in PAST [10]. In addition, dendrogram analysis based on the Bray–Curtis dissimilarity metric was performed. All results were normalized to protein concentration determined with the Bradford method [11]. All assays in this study were independently performed in triplicate.

## 3. Results

When analyzing and comparing the levels of vitamin C in the four bat species, (regardless of the organ), no significant differences were observed (Table 1).

**Figure 1:**
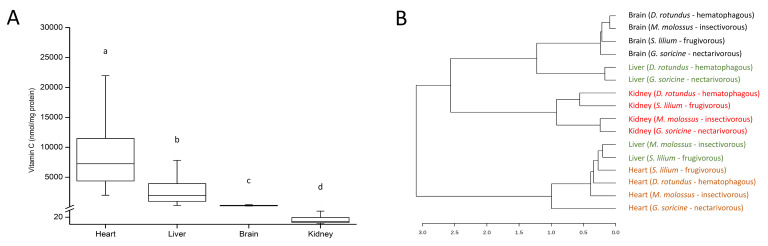
Vitamin C levels in each tissue regardless of the bat species (**A**); error bars represent the standard deviation of the mean; different letters represent statistical significance assessed by PERMANOVA and pairwise test (corrected *p*-value < 0.05). Dendrogram clustering using Bray-Curtis dissimilarity index (**B**); colors were used to differentiate the different tissues.

However, when analyzing and comparing the levels of vitamin C in the four organs (regardless of the species), significant differences were observed (Figure 1). The highest concentrations of vitamin C were measured in the heart, followed by the liver and brain, while the lowest concentration was measured in the kidneys (PERMANOVA, df = 3, MS = 624,765, *F* = 58.61, *p* < 0.0001) (Figure 1A). The dendrogram showed a clear grouping of samples by organ according to the vitamin C levels (Figure 1B); only the liver presented 2 profiles: one grouping with brain samples and the other with the heart. This difference in liver profiles is due to the higher levels of vitamin C in the liver of insectivorous and frugivorous bats, which makes them close to the levels of vitamin C in heart samples.

**Figure 2:**
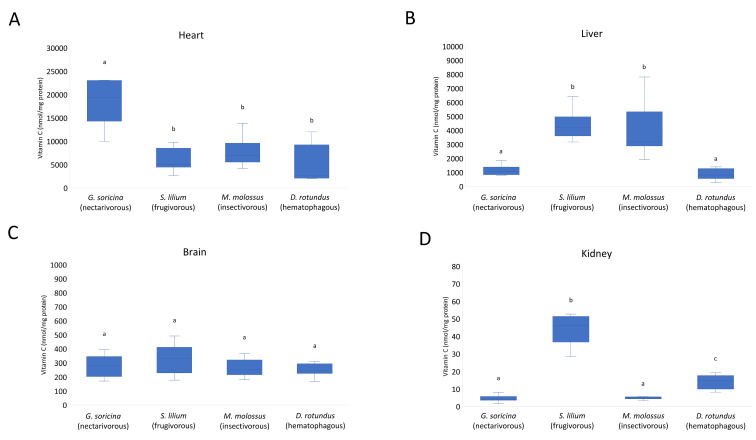
Vitamin C levels in each tissue and bat species. Heart (**A**). Liver (**B**). Brain (**C**). Kidney (**D**). Error bars represent the standard deviation of the mean; different letters represent statistical significance assessed by PERMANOVA and pairwise test (corrected *p*-value < 0.05).

Vitamin C levels in each organ were analyzed and significant differences were observed according to the bat species (PERMANOVA, df = 3, MS = 318,309, *F* = 24.49, *p* < 0.0001) (Figure 2). The heart of the nectarivorous species presented significantly higher levels of vitamin C compared to the other three species (Figure 2A). In the liver, significantly higher levels were observed in frugivorous and insectivorous bats when compared to nectarivorous and hematophagous bats (PERMANOVA, df = 3, MS = 394,172, *F* = 17.55, *p* < 0.0001) (Figure 2B). In the brains of the four bat species, no significant differences were found in the levels of vitamin C (PERMANOVA, df = 3, MS = 13,631, *F* = 1.53, *p* = 0.222) (Figure 2C). In the kidneys, significantly higher levels were observed in the frugivorous species (PERMANOVA, df = 3, MS = 4641, *F* = 33.93, *p* < 0.0001) (Figure 2D).

## 4. Discussion

In this work, we measured and compared vitamin C levels in different organs of four species of adult male bats from different feeding groups (i.e., nectarivorous, frugivorous, insectivorous, and hematophagous). In general, it is expected that the concentration of vitamin C is higher in frugivorous and nectarivorous bats and lower in insectivorous and hematophagous bats. This expectation is primary based on their feeding diets as high concentrations of vitamin C are found mainly in fruits [12] but also in nectar [13,14]. On the other hand, there are still doubts about whether insects synthesize vitamin C, although some studies show the presence of this and other vitamins in Coleoptera, which is the main food of *M. molossus* [15,16,17]. Also, hematophagous bats must acquire vitamin C from the blood plasma of their prey, which is a particularly low source of vitamins. The presence of cattle is common in the region where blood-sucking bats were collected, and we assume that cattle are the food source for these bats.

It was surprising to find no significant differences in the level of vitamin C in these quite distinct bat species. However, when analyzing and comparing the levels of vitamin C in the four organs (regardless of the species), significant differences were observed, which indicates a high degree of metabolic homeostasis. The homeostasis and absorption of vitamin C in the body depends directly on the amount ingested and is regulated by intestinal absorption, tissue accumulation and distribution, utilization and recycling rate, and excretion [18]. The incorporation of this vitamin in tissues is due to sodium–vitamin C transporters (SVCTs) with two isoforms: SVCT 1 and 2. The SVCT2 isoform seems to be the most important for introducing ascorbate into tissues, except in red blood cells [19]. The lethality of SVCT1 and SVCT2 knockout mice reveals the importance of both transporters in vitamin C homeostasis [20].

Additional differences in the levels of vitamin C were only observed when each organ was analyzed according to the species/diet. In this regard, bats are known to have a high metabolism and heart rate [21], so we can assume that the distribution of vitamin C is mainly towards the heart, and even more so in the nectarivorous species since these animals, such as hummingbirds, must maintain flight while feeding [22]. With the high heart rate, oxidative damage could be generated and, to try to prevent or reduce it, the distribution of this vitamin to this vital organ is prioritized.

Liver is the main organ where vitamin C is metabolized and stored, which explains the high rates of this vitamin in the liver. Also, in the areas where the bats were collected, plantations of Moraceae, Bromeliaceae, and Musaceae were observed. Such plantations use pesticides, which may explain the higher levels of vitamin C in the liver of frugivorous and insectivorous. When frugivorous and insectivorous bats feed on the fruits and insects in these plantations, they may be incorporating pesticides, leading to a high burden on the liver, the organ known for detoxifying xenobiotics [23,24]. Vitamin C could be involved in the process of eliminating toxic free radicals and other reactive species, thus benefiting the proper functioning of the liver in these species of bats. [25].

In the brain, vitamin C is an essential molecule. Beyond its antioxidant role, it also has several other important functions, participating as a co-factor in several metabolic pathways [26]. In mammals, the highest concentrations of vitamin C in the body are found in the brain and neuroendocrine tissues [27]. However, the levels of vitamin C in bats were found to be low when compared to the heart and liver. In addition, no difference was found in its levels in the four brain species, indicating a strict regulation on keeping a regular and low concentration of this vitamin in the brain of bats, regardless the type of diet.

The kidneys filter an excess of vitamins from the body, so low levels of vitamin C are expected in this organ. The particularly higher levels of vitamin C in the kidneys of frugivorous may be explained by the high levels of vitamin C in fruits, which may exceed the necessary intake for bats and need to be filtered and eliminated from their body. Indeed, the toxic effect of a high vitamin C supplementation in fruit bats have already been reported [28].

In summary, the fact that these bat species do not present differences in the concentration of vitamin C, despite the marked difference in the type of diet, while keeping a high degree of metabolic homeostasis, leads us to question which stage would be responsible for these intriguing results: Would intestinal uptake be more efficient in animals with diets containing lower concentrations of vitamins? Or would there be transporters present in the tissues? These questions need to be answered in future studies. A better understanding on the body homeostasis of vitamin C may shed new light on the functional roles of this vitamin in animals.

## Figures and Tables

**Table 1:** Pairwise PERMANOVA test among the group of samples grouped according to bat species. No significant difference was observed.

	*G. soricina* (Nectarivorous)	*S. lilium* (Frugivorous)	*M. molossus* (Insectivorous)	*D. rotundus* (Hematophagous)
***G. soricina* (nectarivorous)**		0.3234	0.3708	0.8576
***S. lilium* (frugivorous)**	0.3234		0.9719	0.7154
***M. molossus* (insectivorous)**	0.3708	0.9719		0.4864
***D. rotundus* (hematophagous)**	0.8576	0.7154	0.4864	

## Data Availability

Not applicable.

## Supplementary Materials

The following supporting information can be downloaded at: https://www.mdpi.com/article/10.3390/life12122121/s1. Table S1: Location and coordinates of each sample collection site in Southern Brazil.
