# Supplementary material for: Vitamin C Levels in Different Organs of Bat Species from Different Food Groups
Source: Life (Basel). 2022 Dec 15;12(12):2121. doi: 10.3390/life12122121 (PMC9783699; doi:10.3390/life12122121)
Supplement: Supplementary file 1 [file life-12-02121-s001.zip › life-2027617-supplementary.pdf]

Supplementary Table S1. Location and coordinates of each sample collection site in Southern Brazil.

**Bat Species**	**Food Group**	**n**	**Location (City - State)**	**Coordinates**
G. soricina	Nectarivorous	10	Dom Pedro Alcântara - RS	29°24'22.35"S 49°51'4.56′′W
S. lilium	Frugivorous	10	Dom Pedro Alcântara - RS	29°24'22.35"S 49°51'4.56′′W
M. molossus	Insectivorous	10	Treviso - SC	28°30'47.52"S 49°27'26.6" W
D. rotundus	Hematophagous	9	Criciúma - SC	28°41'27.7"S 49°25'50.6"W
